# Supplementary material for: New multimodal intervention to reduce irritable bowel syndrome (IBS) severity symptoms—Pilot study with a 12 month follow-up
Source: PLoS One. 2022 Nov 21;17(11):e0277880. doi: 10.1371/journal.pone.0277880 (PMC9678296; doi:10.1371/journal.pone.0277880)
Supplement: S1 File — (PDF) [file pone.0277880.s002.pdf]

ENGLISH TRANSLATION

S H A R K – Study

Study of the Havelhöhe Community Hospital on interventions for irritable bowel syndrome in the context of short term therapy

Requested by

Prof. Dr. med. Harald Matthes, Dipl.-Psych. Stefan Dörner
Community Hospital Havelhöhe

In cooperation with the Working Group on Integrative and Anthroposophical Medicine at the Institute for Social Medicine
Institute for Social Medicine, Epidemiology and Health Economics of the Charité - Universitätsmedizin Berlin under the direction of Prof. Dr. med. Harald Matthes.
Dipl.-Psych. Maximilian Hinse

Havelhöhe Community Hospital gGmbH
Clinic for Anthroposophic Medicine
Department of Psychosomatic Medicine and Psychotherapy
Academic teaching hospital of the Charité

Table of contents

1. Introduction and research question 1

2. Presentation of the clinical picture of irritable bowel syndrome 1

Epidemiology 2

Course of the disease 2

Diagnostics 2

3. Presentation of the short-term therapy approach 3

Therapy elements / modules 3

3.1.1. Psychoeducation 3

3.1.2. Artistic therapies (sculpting, painting) 3

Target group and study participants 4

Treatment period – weekly schedule 4

Figure 1 Plan of therapy elements – overview 5

Therapiemanual / Patientenliteratur / Arbeitsblätter .... ***Fehler! Textmarke nicht definiert.***

4. Project design 5

Projektdurchführung .... ***Fehler! Textmarke nicht definiert.***

Timetable and study design 6

Instruments / Test diagnostics 6

Location 8

5. Literature 8

1. Introduction and research question

The following is an overview of the components and procedures of the planned intervention study on irritable bowel syndrome at the Havelhöhe Community Hospital in cooperation with the Max Lüscher Foundation (Switzerland) and the Steinbeis University of Applied Sciences Berlin.

The aim is to investigate the effects (in particular the improvement of physical and mental well-being) of an integrative short-term therapy approach with multimodal therapy elements based on anthroposophic medicine in the treatment of irritable bowel syndrome. For this purpose, different therapy modules and their effects on a cohort of irritable bowel patients will be analysed (1st study hypothesis: therapy effect ~~versus waiting group~~).

Various survey instruments will be used within the study (see point 4.3.). It is to be investigated whether and which schema disorders predispose the irritable bowel syndrome. Here, the Lüscher colour test as a non-verbal personality test is to be compared with validated psychometric tests (2nd study hypothesis: participant collective shows specific uniform patterns / 3rd study hypothesis: survey differences between non-verbal and verbal survey instruments).

2. Presentation of the clinical picture of irritable bowel syndrome

In gastroenterology, irritable bowel syndrome is one of the functional gastrointestinal diseases that are often characterised by typical constellations of symptoms. The following typical symptoms can be present in patients together, individually or in any combination.

- Abdominal pain (abdominal cramps, malaise, flatulence)
- Change in stool behaviour or stool consistency,
- often alternating between constipation and diarrhoea,
- Mucus on the stool,
- Feeling of incomplete bowel emptying (Layer et al., 2011).

Combinations of complaints and symptoms are quite common. In addition to the typical symptoms, there may be

- mental and physical exhaustion as well as
- other psychological symptoms (ibid.).

Epidemiology

The prevalence and incidence of IBS patients depend on the definitions used (Manning, Kruis, Rome I, II, III). According to current studies, the prevalence ranges from 2.5 to 25% (Manning), 5.5 to 13.6% (Rome I) and 2.5 to 19.1% (Rome II). These show a higher prevalence and variability according to Manning compared to Rome I and II criteria. Here, the number of Manning criteria used influences the prevalence from 2.5 - 37%; when 3 Manning criteria are used, the prevalence is about 10%. The pooled prevalence is 7% (Leyer et al., 2011).

Course of the disease

Irritable bowel syndrome regresses spontaneously in some patients, but is often chronic. There is no increased co-prevalence with other serious diseases of the gastrointestinal tract, but certainly with serious other diseases, such as depression, anxiety disorders, somatoform disorders and rigid personality accentuation.

The prognosis of IBS depends on the length of the medical history. Patients with a long medical history are less likely to improve. In this case, permanent life stress is relevant to the prognosis. Irritable bowel syndrome is not associated with the development of other gastrointestinal or other serious diseases and does not have an increased mortality. Irritable bowel patients have a higher risk of surgery (hysterectomy, cholecystectomy) than non-irritable bowel patients.

Diagnostics

According to the currently valid German medical guidelines, irritable bowel syndrome exists if all of the following three conditions are fulfilled. 1:

1. there are chronic complaints, i.e. lasting longer than 3 months (e.g. abdominal pain, flatulence), which are referred to the intestine by the patient and doctor and are usually accompanied by changes in bowel movements.
2. the complaints should justify the patient seeking help and/or worrying about it, and be so severe that the quality of life is relevantly impaired by it.
3. it is a prerequisite that there are no changes characteristic of other clinical pictures which could probably be responsible for these symptoms.

3. Presentation of the short-term therapy approach

Therapy elements / modules

3.1.1. Psychoeducation

Psychoeducation serves to inform patients about the disease and its course as well as a healing way of dealing with it. In particular, the following goals are pursued through the structured communication of information:
- Promotion of the understanding of the disease and the self-responsible handling of it
- Support in coping with the disease
- Reduction of fears, feelings of guilt and shame.

3.1.2. Artistic therapies (sculpting, painting)

Within the art therapy, tried and tested methods are used by experienced therapists from the Havelhöhe Clinic. As "non-verbal psychotherapy", these are particularly effective on the psychological level of the patients and promote introspective self-regulation processes and are also intended to serve as an exercise of will in the study setting.

3.1.3. Movement therapy (eurythmy therapy)

Eurythmy therapy as a special movement therapy is intended to mediate the patient's balance between tension and relaxation and to be activated by rhythmic exercises with the body. Within movement therapy, patients learn to better perceive and evaluate their own bodily processes and to enter into their own process of change.

3.1.4. External application

Within this module, different wraps and their indications are introduced and tried out, which the patients can then apply on their own, easily and inexpensively, if necessary.

3.1.5. Healing Imagination

In the context of healing imagination, an evidence-based, specially developed intestinal hypnosis is used, which is intended to give the patients peace and relaxation and relief from their complaints.

3.1.6. Nutritional counselling

This is to advise patients on specifics of their diet and eating habits. Individual questions, food intolerances and allergies should also be addressed.

3.1.7. Cognitive training group

Within the cognitive training group, the contents of the specific treatment day are reflected on, among other things, with the help of structuring work materials. This is to ensure that the patients deal with the therapy elements in the long term and can also apply individual elements in everyday life.

Target group and study participants

Target group/ Requirements	Requirements Participants who meet the diagnostic criteria according to 2.3.
Group size	A group size of 12-15 participants is planned. This means that with 4 planned groups, a total number of 60 participants is planned.
Contra-indications	Suicidality, psychotic experience, other study participation
Recruitment of participants	Press, waiting list, Internet, GPs, MVZs

Treatment period – weekly schedule

- Within the first four weeks, two treatment days per week and within the second four weeks, one treatment day per week (12 treatment days in total).
- - 2 groups per week: Mon / Wed ; Tue / Thu (16.30 to 18.30 hrs and 19.00 to 21.00 hrs respectively)

**1. Day**	**2. Day**	**3. Day**	**4. Day**	**5. Day**	**6. Day**
Psychoeducation	Movement therapy	Psychoeducation	Movement therapy	Psychoeducation	Movement therapy
Painting	External Application	Painting	External Application	Painting	External Application
Healing Imagination	Nutritional counseling	Healing Imagination	Nutritional counseling	Healing Imagination	Nutritional counseling
Cognitive training group	Cognitive training group	Cognitive training group	Cognitive training group	Cognitive training group	Cognitive training group
**7. Day**	8. Day	9. Day	10. Day	11. Day	12.Day

Psychoeducation	Movement therapy	Psychoeducation	Movement therapy	Psychoeducation	Movement therapy
Sculpting	External Application	Sculpting	External Application	Sculpting	External Application
Healing Imagination	Nutritional counseling	Healing Imagination	Nutritional counseling	Healing Imagination	Nutritional counseling
Cognitive training group	Cognitive training group	Cognitive training group	Cognitive training group	Cognitive training group	Cognitive training group

Figure 1 Plan of therapy elements – overview

Therapy manual / patient literature / worksheets

In order to ensure an equivalent, standardised implementation of the individual therapy elements, the following therapy manuals are to be created:

- Psychoeducation
- External application
- Healing imagination
- Cognitive training group

The contents of the mentioned therapy elements will be planned for the study and documented within the manuals. Furthermore, the patients will receive information material in the form of flyers at the beginning of the therapy on the following modules:

- Psychoeducation
- External application

Within the cognitive training group, work materials will be used to incorporate exercise elements into the therapy. For each of the 12 therapy days, a worksheet is designed to reflect the contents of the day. In addition, patients are given a booklet for self-documentation during and after the 12 days of treatment.

4. Project design

Project implementation

The project is to be carried out by an assembled project team. The following persons are planned for the project team:

- Patient acquisition: Gastroenterology / Psychosomatics secretariat
- Information meeting (preliminary talk)
- Anamnesis interview

- Psychoeducation
- Painting
- Sculpting
- Imaginations
- Nutritional counselling
- Body therapy
- Cognitive training group
- External application

Management and supervision: H. Matthes, S. Dörner (Havelhöhe), Maria Sepke, W. Albert (Steinbeis University Berlin), M. Hinse (Charité).
Follow-up study: student

Timetable and study design

With a planned number of 4 groups and a duration of 8 weeks per group, this results in a pure survey period of 12 weeks (from mid-January 2018 to mid-April 2018). The two waiting groups start the intervention from the 5th week. Patients will be randomly assigned to the groups. Treatment groups 1 and 2 will receive treatment on Mondays and Wednesdays, while waiting groups 3 and 4 will receive treatment on Tuesdays and Thursdays, thus guaranteeing that treatment and waiting groups cannot meet.

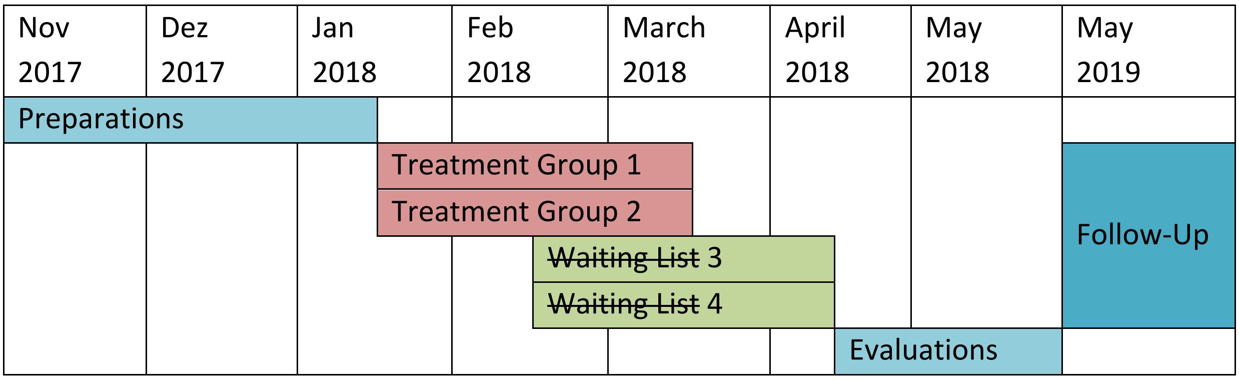

Abbildung 1 Zeitplan des Projektes

Instruments / Test diagnostics

Three survey dates are planned within the framework of a pretest-posttest design with a final follow-up. This is to ensure that differences between pre- and posttest measurements can be collected. A follow-up survey after 12 months will examine the long-term effect of the intervention. Furthermore, two survey dates are planned as part of the comparison between treatment ~~and waiting list group~~. Care should be taken during the survey to ensure that the duration of the tests is reasonable. For this reason, all questionnaires will be sent to the patients

in advance so that they are already pre-filled at the survey dates. During the surveys, only the Lüscher colour test and the SKID-II are then carried out on site. The posttest questionnaires were distributed at the last appointment and completed directly by some patients. The follow-up questionnaires for the 12-month evaluation of the intervention will be sent to patients by post. The follow-up includes two new questionnaires to assess the patients' independent implementation of the exercises and supports learned in the intervention within the last 12 months after the end of the intervention.

Survey time point 1 Screening & pre-test (Dec 2017):

- General anamnesis sheet
- Irritable Bowel Syndrome - Symptom Severity Scale (IBS-SSS)
- Patient Health Questionaires (PHQ-9)
- General Anxiety Disorder (GAD-7)
- Schema Mode Inventory (SMI)
- Lüscher colour test
- Structured Clinical Interview for Personality Disorders (SCID-II)

~~Survey Timepoint 2 (Prätest Treatment Warteliste; Februar 2018)~~

- ~~Irritable Bowel Syndrome – Symptom Severity Scale (IBS-SSS)~~
- ~~Patient Health Questionaires (PHQ-9)~~
- ~~General Anxiety Disorder (GAD-7)~~

Survey period 3 (posttest treatment; March 2018)

- Irritable Bowel Syndrome - Symptom Severity Scale (IBS-SSS)
- Patient Health Questionaires (PHQ-9)
- General Anxiety Disorder (GAD-7)
- Schema Mode Inventory (SMI)
- Questionnaire for the Evaluation of Anthroposophic Therapies (EVAL26)

~~Erhebungszeitraum 4 (Posttest Warteliste 2018):~~

- ~~Irritable Bowel Syndrome – Symptom Severity Scale (IBS-SSS)~~
- ~~Patient Health Questionaires (PHQ-9)~~
- ~~General Anxiety Disorder (GAD-7)~~
- ~~Schema Mode Inventory (SMI)~~
- ~~Fragebogen zur Evaluation der anthroposophischen Therapien (EVAL26)~~

Survey period 5 - (12 months follow-up May 2019)

- General medical history Follow-up
- Irritable Bowel Syndrome - Symptom Severity Scale (IBS-SSS)
- Patient Health Questionaires (PHQ-9)
- General Anxiety Disorder (GAD-7)
- Questionnaire for independent application of anthroposophic therapies

Location

Psychosomatische Tagesklinik GKH / Haus 23 / Kladower Damm 221; 14089 Berlin

5. Literature

Häuser, W. (2008). *Hypnose beim Reizdarm.* In: Revenstorf D, Peter B (Hrsg.): Hypnose in Psychotherapie, Psychosomatik und Medizin. Springer Verlag. 555-568.

Layer, P. et al. (2011). *S3-Leitlinie Reizdarmsyndrom.* AWMF-Registriernummer: 021/016. Z Gastroenterol, Georg Thieme Verlag KG Stuttgart, 237 – 293.

Matthes, H. (2002). Anthroposophische Medizin in Diagnostik und Therapie der funktionellen Darmerkrankungen. Der Merkurstab. Beiträge zur einer Erweiterung der Heilkunst. 2002;55(1):2-11.

Spiller, R., Aziz, Q., Creed, F. et al. (2007). Guidelines on the irritable bowel syndrome: mechanisms and practical management. Gut, 56: 1770–1798.
