# Supplementary material for: New multimodal intervention to reduce irritable bowel syndrome (IBS) severity symptoms—Pilot study with a 12 month follow-up
Source: PLoS One. 2022 Nov 21;17(11):e0277880. doi: 10.1371/journal.pone.0277880 (PMC9678296; doi:10.1371/journal.pone.0277880)
Supplement: S2 File — (PDF) [file pone.0277880.s003.pdf]

Prüfplan

S H A R K – Studie

Studie des Gemeinschaftskrankenhauses Havelhöhe zu Interventionen bei dem Reizdarmsyndrom im Rahmen einer Kurzzeittherapie

Beantragt von

Prof. Dr. med. Harald Matthes, Dipl.-Psych. Stefan Dörner
Gemeinschaftskrankenhaus Havelhöhe

In Kooperation mit der AG Integrative und Anthroposophische Medizin am Institut für Sozialmedizin, Epidemiologie und Gesundheitsökonomie der Charité - Universitätsmedizin Berlin unter der Leitung von Prof. Dr. med. Harald Matthes.
Dipl.-Psych. Maximilian Hinse

Gemeinschaftskrankenhaus Havelhöhe gGmbH
Klinik für Anthroposophische Medizin
Abteilung für Psychosomatische Medizin und Psychotherapie
Akademisches Lehrkrankenhaus der Charité

Inhaltsverzeichnis

1. Einführung & Fragestellung **1**

2. Darstellung des Krankheitsbildes Reizdarmsyndrom **1**
Epidemiologie *2*
Krankheitsverlauf *2*
Diagnostik *2*

3. Darstellung des Kurzzeit-Therapieansatzes **3**
Therapie-Elemente /-Module *3*
3.1.1. Psychoedukation 3
3.1.2. Künstlerische Therapien (Plastizieren, Malen) 3
3.1.3. Bewegungstherapie (Heileurythmie) 3
3.1.4. Äußere Anwendung 3
3.1.5. Heilsame Imagination 3
3.1.6. Ernährungsberatung 4
3.1.7. Kognitive Trainingsgruppe 4
Zielgruppe und StudienteilnehmerInnen *4*
Behandlungszeitraum – Wochenplan *4*
Therapiemanual / Patientenliteratur / Arbeitsblätter *5*

4. Projektdesign **5**
Projektdurchführung *5*
Zeitplan und Untersuchungsdesign *6*
Erhebungsinstrumente / Testdiagnostik *6*
Standort *7*

5. Literatur **8**

1. Einführung & Fragestellung

Im Folgenden soll ein Überblick gegeben werden über die Bestandteile und Abläufe der geplanten Interventionsstudie zum Reizdarmsyndrom am Gemeinschaftskrankenhaus Havelhöhe in Kooperation mit der Max Lüscher Stiftung (Schweiz) und der Steinbeis Hochschule Berlin.

Ziel ist es Auswirkungen (insbesondere die Verbesserung des körperlichen und seelischen Befindens) eines integrativen Kurzzeit-Therapieansatzes mit multimodalen Therapieelementen auf der Grundlage der Anthroposophischen Medizin bei der Behandlung des Reizdarmsyndroms zu untersuchen. Hierzu sollen verschiedene Therapiemodule und deren Wirkungen auf eine Kohorte von Reizdarm-Patienten analysiert werden (1. Studienhypothese: Therapiewirkung ~~versus Wartegruppe~~).

Innerhalb der Studie werden verschiedene Erhebungsinstrumente angewandt (siehe Punkt 4.3.). Es soll untersucht werden, ob und welche Schemastörungen das Reizdarmsyndrom prädestinieren. Hierbei soll der Lüscher Farbtest als non-verbaler Persönlichkeitstest mit validierten psychometrischen Tests verglichen werden (2. Studienhypothese: Teilnehmerkollektiv weist spezifische einheitliche Muster auf / 3. Studienhypothese: Erhebungsdifferenzen zwischen non-verbalen und verbalen Erhebungsinstrumenten).

2. Darstellung des Krankheitsbildes Reizdarmsyndrom

Das Reizdarmsyndrom zählt in der Gastroenterologie zu den funktionellen Magen-Darm-Erkrankungen, die sich häufig durch typische Symptomkonstellationen auszeichnen. Die nachfolgenden typischen Symptome können zusammen, einzeln oder in beliebiger Kombination bei den Patienten vorhanden sein.

- Bauchschmerzen (Bauchkrämpfe, Unwohlsein, Blähungen)
- Veränderung des Stuhlverhaltens bzw. der Stuhlbeschaffenheit,
- häufig auch Wechsel zwischen Verstopfung und Durchfall,
- Schleim auf dem Stuhl,
- Gefühl der unvollständigen Darmentleerung (Layer et al., 2011).

Kombinationen der Beschwerden und Symptome sind dabei recht häufig. Zu den typischen Symptomen können hinzukommen:

- geistige und körperliche Erschöpfung sowie
- andere psychische Symptome (ebd.).

Epidemiologie

Die Prävalenz und Inzidenz von Reizdarm-Patienten sind abhängig von den benutzen Definitionen (Manning, Kruis, Rom I, II, III). Nach aktuellen Studien liegt die Prävalenz zwischen 2,5 und 25% (Manning), 5,5 und 13,6% (Rom I) und 2,5 und 19,1% (Rom II). Diese zeigen eine höhere Prävalenz und Variabilität nach den Manning im Vergleich zu Rom-I- und -II-Kriterien. Hierbei beeinflusst die Anzahl der verwendeten Manning-Kriterien die Prävalenz von 2,5 – 37%, bei Verwendung von 3 Manning-Kriterien liegt die Prävalenz bei ca. 10%. Die gepoolte Prävalenz liegt bei 7% (Leyer et al., 2011).

Krankheitsverlauf

Das Reizdarmsyndrom ist bei einem Teil der Patienten spontan rückläufig, häufig aber auch chronisch verlaufend. Es besteht keine gesteigerte Koprävalenz mit anderen schwerwiegenden Erkrankungen des Gastrointestinaltrakts, aber durchaus mit schwerwiegenden anderen Erkrankungen, wie z. B. Depression, Angststörungen, somatoformen Störungen und starren Persönlichkeitsakzentuierungen.

Die Prognose des Reizdarmsyndroms ist abhängig von der Länge der Krankengeschichte. Patienten mit einer langen Krankengeschichte weisen eine geringere Wahrscheinlichkeit der Besserung auf. Hierbei ist ein permanenter Lebensstress prognoserelevant. Das Reizdarmsyndrom ist nicht mit der Entwicklung anderer gastrointestinaler oder anderer schwerwiegender Erkrankungen assoziiert und weist keine erhöhte Mortalität auf. Reizdarmpatienten haben ein höheres Risiko operiert zu werden (Hysterektomie, Cholezystektomie) als Nicht-Reizdarmpatienten.

Diagnostik

Nach den derzeit gültigen medizinischen deutschen Leitlinien liegt ein Reizdarmsyndrom vor, wenn alle der drei nachfolgenden Bedingungen erfüllt sind:

1. Es bestehen chronische, d. h. länger als 3 Monate anhaltende Beschwerden (z. B. Bauchschmerzen, Blähungen), die von Patient und Arzt auf den Darm bezogen werden und in der Regel mit Stuhlgangsveränderungen einhergehen.
2. Die Beschwerden sollen begründen, dass der Patient deswegen Hilfe sucht und/oder sich sorgt, und so stark sein, dass die Lebensqualität dadurch relevant beeinträchtigt wird
3. Voraussetzung ist, dass keine für andere Krankheitsbilder charakteristischen Veränderungen vorliegen, welche wahrscheinlich für diese Symptome verantwortlich sein könnten.

3. Darstellung des Kurzzeit-Therapieansatzes

Therapie-Elemente /-Module

3.1.1. Psychoedukation

Die Psychoedukation dient der Aufklärung der Patienten über die Krankheit und ihren Verlauf sowie einen heilsamen Umgang damit. Insbesondere werden durch eine strukturierte Vermittlung von Informationen folgende Ziele verfolgt:

- Förderung des Krankheitsverständnisses und des selbstverantwortlichen Umgangs
- Unterstützung bei der Krankheitsbewältigung
- Reduktion von Ängsten, Schuld- & Schamgefühlen

3.1.2. Künstlerische Therapien (Plastizieren, Malen)

Innerhalb der Kunsttherapie werden erprobte Verfahren von erfahrenen Therapeuten aus dem Klinikum Havelhöhe angewandt. Diese setzen als „nonverbale Psychotherapie“ insbesondere auf der seelischen Ebene der Patienten an und fördern introspektive Selbstregulationsprozesse und sollen in dem Studiensetting auch als Willensübung dienen.

3.1.3. Bewegungstherapie (Heileurythmie)

Die Heileurythmie als spezielle Bewegungstherapie soll das Gleichgewicht des Patienten zwischen Anspannung und Entspannung vermitteln und durch rhythmische Übungen mit dem Körper aktiviert werden. Die Patienten lernen innerhalb der Bewegungstherapie eigene Körperprozesse besser wahrzunehmen, zu bewerten und in einen eigenen Veränderungsprozess zu kommen.

3.1.4. Äußere Anwendung

Innerhalb dieses Moduls werden verschiedenen Wickel und ihre Indikationen vorgestellt und ausprobiert, die die Patienten anschließend bei Bedarf einfach und kostengünstig eigenständig anwenden können.

3.1.5. Heilsame Imagination

Im Rahmen der Heilsamen Imagination wird eine evidenzbasierte speziell entwickelte Darmhypnose angewandt, welche den Patienten Ruhe und Entspannung und Linderung Ihrer Beschwerden vermitteln soll.

3.1.6. Ernährungsberatung

Hier sollen die Patienten auf Spezifika im Rahmen ihrer Ernährung und Essgewohnheiten hingewiesen werden. Es soll auch auf individuelle Fragen, Nahrungsmittelunverträglichkeiten und Allergien eingegangen werden.

3.1.7. Kognitive Trainingsgruppe

Innerhalb der Kognitiven Trainingsgruppe werden u.a. anhand von strukturierenden Arbeitsmaterialien die Inhalte des spezifischen Behandlungstages reflektiert, siehe auch Punkt 3.4. Hierdurch soll sichergestellt werden, dass die Patienten sich mit den Therapieelementen nachhaltig auseinandersetzen und einzelne Elemente auch im Alltag anwenden können.

Zielgruppe und StudienteilnehmerInnen

Zielgruppe / Voraussetzungen	TeilnehmerInnen, die die diagnostischen Kriterien gemäß 2.3. aufweisen.
Gruppengröße	Geplant ist eine Gruppengröße von 12-15 TeilnehmerInnen. D.h. bei 4 geplanten Gruppen ist eine Gesamtteilnehmerzahl von 60 Personen geplant.
Kontra-Indikation	Suizidalität, psychotisches Erleben, anderweitige Studienteilnahme
Rekrutierung der Patienten	Presse, Warteliste, Internet, Hausärzte, MVZ

Behandlungszeitraum – Wochenplan

- Innerhalb der ersten vier Wochen, zwei Behandlungstage pro Woche und innerhalb der zweiten vier Wochen ein Behandlungstag pro Woche (insgesamt 12 Behandlungstage)
- 2 Gruppen pro Woche: Mo / Mi ; Di / Do (16.30 bis 18.30 Uhr bzw. 19.00 bis 21.00 Uhr)

1. Tag	2. Tag	3. Tag	4. Tag	5. Tag	6. Tag
Psychoedukation	Bewegungs-therapie	Psychoedukation	Bewegungs-therapie	Psychoedukation	Bewegungs-therapie
Malen	Äußere Anwendung	Malen	Äußere Anwendung	Malen	Äußere Anwendung
Heilsame Imagination	Ernährungs-beratung	Heilsame Imagination	Ernährungs-beratung	Heilsame Imagination	Ernährungs-beratung
Kognitive Trainingsgruppe	Kognitive Trainingsgruppe	Kognitive Trainingsgruppe	Kognitive Trainingsgruppe	Kognitive Trainingsgruppe	Kognitive Trainingsgruppe
**7. Tag**	**8. Tag**	**9. Tag**	**10. Tage**	**11. Tag**	**12. Tag**
Psychoedukation	Bewegungs-therapie	Psychoedukation	Bewegungs-therapie	Psychoedukation	Bewegungs-therapie
Malen	Äußere Anwendung	Plastizieren	Plastizieren	Plastizieren	Plastizieren
Heilsame Imagination	Ernährungs-beratung	Heilsame Imagination	Heilsame Imagination	Heilsame Imagination	Heilsame Imagination

Kognitive Trainingsgruppe	Kognitive Trainingsgruppe	Kognitive Trainingsgruppe	Kognitive Trainingsgruppe	Kognitive Trainingsgruppe	Kognitive Trainingsgruppe

Abbildung 1 Plan der Therapieelemente - Überblick

Therapiemanual / Patientenliteratur / Arbeitsblätter

Um eine gleichwertige, standardisierte Durchführung der einzelnen Therapieelemente zu gewährleisten, sollen folgende Therapiemanuale erstellt werden:

- Psychoedukation
- Äußere Anwendung
- Heilsame Imagination
- Kognitive Trainingsgruppe

Die Inhalte der genannten Therapieelemente werden für die Studie geplant und innerhalb der Manuale festgehalten. Weiterhin sollen die Patienten Informationsmaterial in Form von Flyern zu Beginn der Therapie zu folgenden Modulen erhalten:

- Psychoedukation
- Äußere Anwendung

Innerhalb der *Kognitiven Trainingsgruppe* werden Arbeitsmaterialien genutzt um übende Elemente in die Therapie einzubeziehen. Für jeden der 12 Therapietage wird hierzu ein Arbeitsblatt entworfen, welches die Inhalte des Tages reflektiert. Zusätzlich wird den Patienten ein Heft zur Selbstdokumentation während und nach den 12 Behandlungstagen mitgegeben.

4. Projektdesign

Projektdurchführung

Das Projekt soll durch ein zusammengestelltes Projektteam durchgeführt werden. Für das Projektteam sind folgende Personen geplant:

- Patienten-Akquise: Sekretariat Gastroenterologie / Psychosomatik
- Info-Veranstaltung (Vorgespräch): Stefan Dörner
- Anamnese-Gespräch:
  (Studierende Steinbeis Hochschule Berlin)
- Psychoedukation:
- Malen:
- Plastizieren:
- Imaginationen:
- Ernährungsberatung:
- Körpertherapie:
- Kognitive Trainingsgruppe:

- Äußere Anwendung: Anna Kruschel, Mathias Hunklinger

Leitung und Supervision: H. Matthes, S. Dörner (Havelhöhe), Maria Sepke, W. Albert (Steinbeis Hochschule Berlin), M. Hinse (Charité).
Follow-Up Untersuchung: (Steinbeis Hochschule Berlin).

Zeitplan und Untersuchungsdesign

Bei einer geplanten Anzahl von 4 Gruppen und einer Dauer von 8 Wochen pro Gruppe ergibt sich ein reiner Erhebungs-Zeitraum von 12 Wochen (von Mitte Januar 2018 bis Mitte April 2018). Die zwei Wartegruppen beginnen ab der 5. Woche mit der Intervention. Die Patienten werden zufällig in die Gruppen eingeteilt. Die Treatment Gruppen 1 und 2 erhalten die Behandlung Montags und Mittwochs, die Warteliste Gruppen 3 und 4 Dienstags und Donnerstag, damit wird garantiert, dass Treatment- und Wartegruppen sich nicht begegnen können.

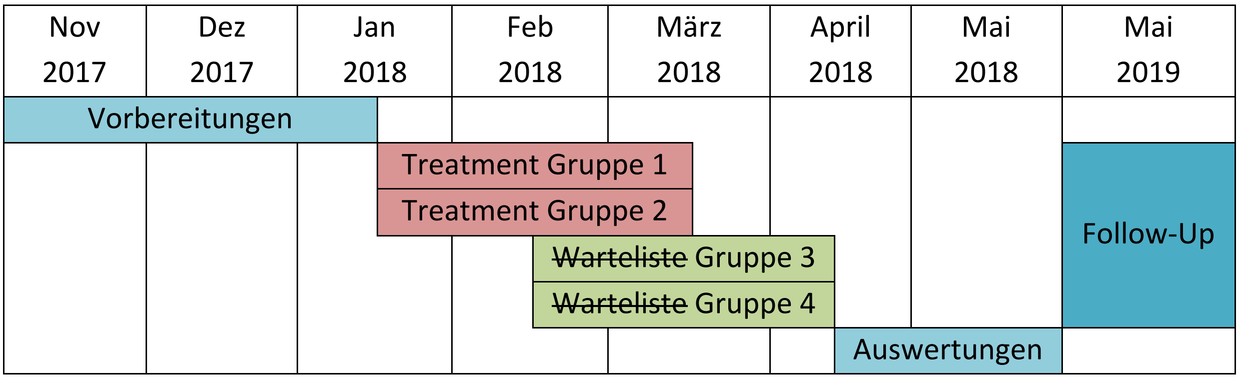

Abbildung 2 Zeitplan des Projektes

Erhebungsinstrumente / Testdiagnostik

Geplant sind drei Erhebungstermine im Rahmen eines Prätest-Posttest-Designs mit abschließendem Follow-Up. Somit soll sichergestellt werden, dass Unterschiede zwischen Prä- und Postmessung erhoben werden können. Eine Follow-Up Erhebung nach 12 Monaten soll den langfristigen Effekt der Intervention Untersuchen. ~~Weiterhin sind zwei Erhebungstermine im Rahmen des Vergleichs zwischen Treatment und Wartelistgruppe geplant~~. Es soll bei der Erhebung darauf geachtet werden, dass die Zumutbarkeit der Dauer der Tests gewährleistet ist. Aus diesem Grund werden alle Fragebögen vorab an die Patienten verschickt, sodass diese zu den Erhebungszeitpunkten bereits vorausgefüllt sind. Bei den Erhebungen werden dann vor Ort nur der Lüscher-Farbtest und der SKID-II durchgeführt. Die Posttest Fragebögen wurden zum letzten Termin ausgeteilt und von einigen Patienten direkt ausgefüllt. Die Follow-Up Fragebögen zur 12 monatigen Evaluation der Intervention werden per Post an die Patienten versendet. Das Follow-Up beinhaltet zwei neue Fragebögen die die eigenständige Umsetzung der in der Intervention erlernten Übungen und Hilfestellungen durch die Patienten innerhalb der letzten 12 Monate nach Abschluss der Intervention untersuchen sollen.

Erhebungszeitpunkt 1 Screening & Prätest (Dez 2017):

- Allgemeiner Anamnesebogen
- Irritable Bowel Syndrome – Symptom Severity Scale (IBS-SSS)
- Patient Health Questionaires (PHQ-9)
- General Anxiety Disorder (GAD-7)
- Schema Mode Inventory (SMI)
- Lüscher-Farbtest
- Strukturiertes Klinisches Interview für Persönlichkeitsstörungen (SKID-II)

~~Erhebungszeitpunkt 2 (Prätest Treatment Warteliste; Februar 2018)~~

- ~~Irritable Bowel Syndrome – Symptom Severity Scale (IBS-SSS)~~
- ~~Patient Health Questionaires (PHQ-9)~~
- ~~General Anxiety Disorder (GAD-7)~~

Erhebungszeitpunkt 3 (Posttest Treatment; März 2018)

- Irritable Bowel Syndrome – Symptom Severity Scale (IBS-SSS)
- Patient Health Questionaires (PHQ-9)
- General Anxiety Disorder (GAD-7)
- Schema Mode Inventory (SMI)
- Fragebogen zur Evaluation der anthroposophischen Therapien (EVAL26)

~~Erhebungszeitraum 4 (Posttest Warteliste 2018):~~

- ~~Irritable Bowel Syndrome – Symptom Severity Scale (IBS-SSS)~~
- ~~Patient Health Questionaires (PHQ-9)~~
- ~~General Anxiety Disorder (GAD-7)~~
- ~~Schema Mode Inventory (SMI)~~
- ~~Fragebogen zur Evaluation der anthroposophischen Therapien (EVAL26)~~

Erhebungszeitraum 5 – (12 Monate Follow-Up Mai 2019)

- Allgemeiner Anamnesebogen Follow-Up
- Irritable Bowel Syndrome – Symptom Severity Scale (IBS-SSS)
- Patient Health Questionaires (PHQ-9)
- General Anxiety Disorder (GAD-7)
- Fragebogen zur selbständigen Anwendung der anthroposophischen Therapien

Standort

Psychosomatische Tagesklinik GKH / Haus 23 / Kladower Damm 221; 14089 Berlin

5. Literatur

Häuser, W. (2008). *Hypnose beim Reizdarm.* In: Revenstorf D, Peter B (Hrsg.): Hypnose in Psychotherapie, Psychosomatik und Medizin. Springer Verlag. 555-568.

Layer, P. et al. (2011). *S3-Leitlinie Reizdarmsyndrom.* AWMF-Registriernummer: 021/016. Z Gastroenterol, Georg Thieme Verlag KG Stuttgart, 237 – 293.

Matthes, H. (2002). *Anthroposophische Medizin in Diagnostik und Therapie der funktionellen Darmerkrankungen. Der Merkurstab.* Beiträge zur einer Erweiterung der Heilkunst. 2002;55(1):2-11.

Spiller, R., Aziz, Q., Creed, F. et al. (2007). *Guidelines on the irritable bowel syndrome: mechanisms and practical management.* Gut, 56: 1770–1798.
