# Supplementary material for: New multimodal intervention to reduce irritable bowel syndrome (IBS) severity symptoms—Pilot study with a 12 month follow-up
Source: PLoS One. 2022 Nov 21;17(11):e0277880. doi: 10.1371/journal.pone.0277880 (PMC9678296; doi:10.1371/journal.pone.0277880)
Supplement: Attachment — Submitted filename: Response to Reviewers.pdf [file pone.0277880.s005.pdf]

Response to Reviewers – Manuscript PONE-D-22-06021

Title: "New multimodal intervention to reduce Irritable Bowel Syndrome (IBS) severity symptoms - pilot study with a 12 month's follow up"

Additional editorial requirements:

1. Please ensure that your manuscript meets PLOS ONE's style requirements, including those for file naming. The PLOS ONE style templates can be found at https://journals.plos.org/plosone/s/file?id=wjVg/PLOSOne_formatting_sample_main_body.pdf and https://journals.plos.org/plosone/s/file?id=ba62/PLOSOne_formatting_sample_title_authors_affiliations.pdf

ANSWER: We went through the PLOS ONE style requirements again and, among other things, reformatted the headings, renamed the Figures to Fig, renamed the tables, and adjusted the file names according to these requirements.

2. We note that the grant information you provided in the 'Funding Information' and 'Financial Disclosure' sections do not match.
   When you resubmit, please ensure that you provide the correct grant numbers for the awards you received for your study in the 'Funding Information' section

ANSWER: We have taken another look at the funding information. Our study has been indirectly funded by the endowed professorship of Harald Matthes, since the participating staff members (MH, AT and HM) are financed by the endowed professorship. However, there is no grant number available or the like. The study has been financed by the resources of the endowed professorship. In our opinion, the "funding information" is correct. We revised the "financial disclosure" and "competing interests" sections and attached the new version at the end of the cover letter.

3. The in-house editorial staff feels that your study meets the World Health Organization definition of a clinical trial because it is a prospective study in which participants received 12 session of AMT to investigate the effects on IBS.

ANSWER: Due to this assessment, the manuscript has already been changed during the editorial process. A CONSORT flow-chart (Fig1) has been used and the TREND checklist has been applied. We hope that this is correct. We also added the registry name and number in the abstract (drks.de, DRKS00016890).

REVIEWER 1:

<u>Important note:</u> This review pertains only to 'statistical aspects' of the study and so 'clinical aspects' [like medical importance, relevance of the study, 'clinical significance and implication(s)' of the whole study, etc.] are to be evaluated [should be assessed] separately/independently. Further please note that any 'statistical review' is generally done under the assumption that (such) study

specific methodological [as well as execution] issues are perfectly taken care of by the investigator(s). This review is not an exception to that and so does not cover clinical aspects {however, seldom comments are made only if those issues are intimately / scientifically related & intermingle with 'statistical aspects' of the study}. Agreed that 'statistical methods' are used as just tools here, however, they are vital part of methodology [and so should be given due importance].

COMMENTS:

1. Please note that, your ABSTRACT is well drafted but assay type. It is preferable [refer to item 1b of CONSORT checklist 2010: Structured summary of trial design, methods, results, and conclusions] to divide the ABSTRACT with small sections like 'Objective(s)', 'Methods', 'Results', 'Conclusions', etc. which is an accepted practice of most of the good/standard journals [including this one, though 'The PLoS One Guidelines to Authors' did not specify an Abstract format, it is desirable]. It will definitely (generally) be more informative then, I guess, whatever the article type may be.

ANSWER: We have revised the abstract and added subheadings accordingly. As our study is not an RCT, the TREND checklist was used in this manuscript (which is also recommended in the PLoSguidelines for non-RCTs). Please find thechecklist as supplementary material in the submission.

2. This study being 'pilot' in nature, sample size is not a big issue. However, [though many things are ignored (loosely looked at / evaluated)] methodological issues need to be very rigorous followed{in case of clinical trial, CONSORT guidelines are to be strictly observed/followed}.

ANSWER: To follow methodological issues and best practice, we used the TREND Checklist, which we followed carefully, please see our previous answer. The checklist is also included as a supplementary material in the submission.

3. I am sure that the authors are aware of the well-known drawbacks of a single-arm design [a type of Quasi-experimental research], and it is often said that 'alright to have 'single-arm design' (before-after study) when that is the only possibility', however, it is very essential to keep the limitations in mind while interpreting results. Considering 'pilot' (a feasibility study) nature of the study although it is acceptable/alright, as you know, a classical/ideal clinical trial/study needs/requires a concurrently {but similarly} handled/treated appropriately selected/chosen control/comparison parallel group/arm. In this context, please read the following which is pasted from one standard textbook on 'Research Methodology':

   "Inferential statistics (i.e., hypothesis testing + estimation of CI) is built on the population model [which means the underlying assumption is that there is/are population(s) and we are dealing with random sample(s) drawn from that/those population(s)]. Although in clinical trial (involving at least two groups) we do not really deal with random samples (generally a non-probabilistic convenience sampling), 'allocation' to treatment groups is 'randomly' done which enable us to evoke the population model and we can use inferential statistics safely. But when there is only one group (so that there is no question of random allocation), with 'non-random' selection, it may be questionable to use inferential statistics even if you have two measurement sets as 'pre-post' or use 'internal grouping for comparison."

   Therefore, I guess, it would have been nice to adhered to original plan [lines 89-90] of 'a waiting group design'.

ANSWER: Thank you very much for pointing this out. Unfortunately, it became apparent during recruitment that the patient group was not so easy to enroll for this setting of therapy. The groups in the evening, for example, were less well received than those in the afternoon. Here it was not possible to offer more or larger groups earlier in the day due to the ongoing hospital operations and the people involved here. Which is why it was not possible to do otherwise than forego a control group. Even in a pilot/feasibility study, it would have been better indeed to have a control group. We added this paragraph in the manuscript for transparency and clarity (line 112). In the discussion, the topic is also taken up in the interpretation of the results (see line 477ff). We hope you agree with the discussion on this.

4. Although all statistical analysesare performed correctly, few table values [example: q-values (false discovery rate correction for multiple testing) in table-2]] are not explained adequately, in my opinion. How to read [and interpret] these table values may be very briefly explained. Same with table-3.

ANSWER: Thank you very much for this helpful hint. We have added further information and explanations in the legend of tables 2 and 3 and in the methods section (line 199ff) for better comprehensibility of q-values.

5. Please note that the measures/tools used are definitely appropriate, but most of them yield data that are in [at the most] ‘ordinal’ level of measurement [and not in ratio level of measurement for sure. Then application of suitable non-parametric test(s) is/are indicated/advisable [even if distribution may be ‘Gaussian’ (i.e. normal)]. Agreed that there is/are no non-parametric test(s)/technique(s) available to be used as alternative in all

situation(s) [suitable / most desired/applicable], but should be used whenever/wherever they are available [for example, instead of paired ‘t’ test use Wilcoxon’s Signed Ranked test].

ANSWER: Thank you very much for your comment. Weagree, non-parametric tests are preferable to parametric ones in most cases. However, even though this study was a pilot/feasibility study with a small sample, we carefully addressed common prior assumptions for our outcomes, e.g. linearity, homogeneity of variances, analysis of normal distribution of residuals and for analysis of outliers, e.g. using analysis plots and results (Field et al., 2012; Field & Wilcox, 2017; Field & Wright, 2011; Lüdecke et al., 2021). In our main analysis, a linear mixed model was calculated in which the assumptions of regressions also apply and were therefore taken into account. Other of these assumptions, such as the assumption of the independence of the errors or the homogeneity of the regression slopes, were balanced by multilevel models (Field & Wright, 2011). We added these informations in the statistics section of Methodology (line 208).

6. Moreover, limitations (if any) of the study are not mentioned/listed anywhere. Does that mean {according to authors} there are none? As pointed out in ‘important note’ above “This review pertains only to ‘statistical aspects’ of the study and so ‘clinical aspects’ should be assessed separately/independently [one should carefully consider/look at the clinical implications of the study].

ANSWER: Limitations of the study were listed and discussed in the discussion. Due to the good advice to also consider the statistical assumptions (comment 5), we have also added more details in the statistics section (see line 196ff).

7. There is no problem in accepting this article after minor revision, in my opinion.

Thank you very much for your valuable feedback and taking time in reviewing the manuscript.

Reviewer #2:

1. The study population should be more specifically characterized so that we could know what the target population of AMT would be. What are the predominant symptoms? How about the subgroups (IBS-C, IBS-D, mixed…)? Did these bowel symptoms improve after AMT?

ANSWER: Thank you for your comment. Unfortunately, no IBS subtypes were recorded in this study and therefore they could not be compared with each other. Furthermore, since this is a pilot study with a small sample, the subtypes were not analysed. In Table 1 and 2, the collected patient characteristics can be seen. The focus of the study was on the diagnosis and differentiation between IBS patients with and without mental comorbidity and its influence on treatment success. The target population has been discussed here under these aspects of mental comorbidity.

2. In this study, female patients were predominant (92% in abstract and 89% in main text, please clarify) so the generalizability of this study should be discussed in more depth. Traditionally, male IBS patients tend to have diarrhea (IBS-D) and female patients are more likely to have constipation.

ANSWER: Thank you for pointing out the typo in the abstract. The number of 89% women is correct and the abstract has been corrected. In addition, the discussion has been adjusted and the overrepresentation of women in this study has been pointed out (see line 474ff).

3. The novelty of this study is AMT. However, several of the modules and sessions of AMT were conducted by psychology students. Did these students receive related medical training regarding IBS? Did they conduct the AMT under surveillance? What were their experiences and background? It would be better to clarify these points before AMT is put into clinical practice.

ANSWER: Thank you very much for pointing this out. The AMT modules were conducted either by young psychologists with a degree under supervision or by experienced therapists und nutritionists from the community hospital. The cooperation of the young psychologists took place during their studies at Steinbeis University as part of their master's thesis and were supervised by experienced therapists from the hospital Gemeinschaftskrankenhaus Havelhöhe.This has been added to the manuscript to make it easier to understand the involvement of the young psychologists under supervision and the cooperation with the professional and experienced therapists from the community hospital (line 131).

4. Similarly, the nutritional counselling and education for food tolerances were conducted by “experienced therapist”. Who were they? Background of nutrition?

ANSWER: A nutritionist with many years of experience at the community hospital also provided nutritional counselling as part of the study. We have added this information in the methods section.

5. Did these patients receive medical treatment during the AMT period? One patient received treatment for psychosis on enrolment? Should this patient be excluded?

ANSWER: Thank you very much for this point. Unfortunately, a person with self-reported psychosis is still listed in the sample description by mistake. The exclusion criteria (line 109) were " lack of diagnosis, suicidal tendencies, psychotic experiences and participation in other studies. Drug therapy for IBS had to be stable or without effect for 6 weeks." The exclusion of the person from the studyhas been added to the flowchart (exclusion criteria). Some of the patients also received medication during the short-term therapy of the study. Of course, they had to continue taking this medication.

6. Since only 21 of the 36 patients had the 12-month follow-up, it may not be very confident to say the AMT effect to be profound and lasting.

ANSWER: Thank you for your comment. We think that the statement applies to these remaining 21 patients, but of course no statement can be made about the other 15 patients who did not respond in the follow-up. We have reformulated the statement in the conclusion and made it more precise.

7. English editing by a native speaker is suggested.

ANSWER: English Proofreading and editing has been performed by a native speaker. Thank you very much for the feedback and your time.

Reviewer #3:

1. Irritable bowel syndrome treatment is various and broad. It means that efficacy of single irritable bowel syndrome treatment is inadequate. Multimodal therapy to improve the severity of IBS in patients without psychological comorbidities with a sustained effect at 12 months' follow-up in this study.

   However there are some biases. First, apart from small sample size, the gender composition is not equilibrium and up to 92% female. Second, AMT was evaluated as a therapy package including physician-patient consultations, the question of specific therapy effects versus non-specific effects (placebo effects, context effects, patient expectations etc.) was not an issue of the present analysis. Third, 51 patients were included in the study but the dropout rate was 29.4%(15/51).

ANSWER: Thank you for your feedback and for taking the time to look at the manuscript. We agree with the comment on the different biases. The sample size is small. Likewise, the gender ratio is not balanced, whereby the prevalence in some cases is also as high as 2:1 women to men. However, as you also note, a therapy limited to a single modality is insufficient for IBS, which is why a multimodal concept was tested for this study. The hurdle here, of course, is that no separate conclusions about the effectiveness of the individual modalities can be made. The focus of the study was to test the therapy concept for future studies and to differentiate the effectiveness of the concept for patients with and without additional mental comorbidity.

NOTE regarding Figures and PACE:

We used the service PACE as suggested for all 3 figures during this revision. We renamed the Figures to Fig1 to Fig3 accordingly.

References:

Field, A. P., Miles, J., & Field, Z. (2012). *Discovering statistics using R*. SAGE Publications, Inc.

Field, A. P., & Wilcox, R. R. (2017). Robust statistical methods: A primer for clinical psychology and experimental psychopathology researchers. *Behaviour Research and Therapy*, *98*, 19–38. https://doi.org/10.1016/j.brat.2017.05.013

Field, A. P., & Wright, D. B. (2011). A Primer on Using Multilevel Models in Clinical and Experimental Psychopathology Research. *Journal of Experimental Psychopathology*, *2*(2), 271–293. https://doi.org/10.5127/jep.013711

Lüdecke, D., Ben-Shachar, M. S., Patil, I., Waggoner, P., & Makowski, D. (2021). performance: An R package for assessment, comparison and testing of statistical models. *Journal of Open Source Software*, *6*(60), 3139. https://doi.org/10.21105/joss.03139
