# Supplementary material for: New multimodal intervention to reduce irritable bowel syndrome (IBS) severity symptoms—Pilot study with a 12 month follow-up
Source: PLoS One. 2022 Nov 21;17(11):e0277880. doi: 10.1371/journal.pone.0277880 (PMC9678296; doi:10.1371/journal.pone.0277880)
Supplement: Attachment — Submitted filename: Responese to reviewers.pdf [file pone.0277880.s006.pdf]

Response to reviewers

Reviewer #4: The manuscript demonstrated an 8-week AMT therapy would improve the IBS and maintain its effect at after 12 months' follow-up, which is limited in the IBS patients without psychological comorbidities. The manuscript has been received one round review by 3 reviewers. And I found the manuscript is interesting and the authors have adequately responded to the 3 reviewers. Nevertheless, I still have some other comments which need to be clarified before final publication.

1. The manuscript should be edited by a native English speaker before publication.

ANSWER: Thank you for this suggestion. The manuscript has now been edited by a native speaker..

2. For most readers, even among IBS specialists, are unfamiliar with anthroposophic therapy. More detailed introduction regarding the background, rationale and efficacy in treating other disorders by using such therpay should be described in the INTRODUCTION.

ANSWER: Thank you for this suggestion. In order to describe the anthroposophic medicine and the concept behind the therapy of irritable bowel syndrome more comprehensibly for the readers, a subheading and an extended passage on anthroposophic medicine has been inserted from line 77 onwards.

3. As described in the Method, the AMT included psychoeducation, psychotherapy, art therapy, external application, and nutritional counselling. What is the rationale for including external application for IBS? What kind of nutrition suggestion was applied in the AMT? A more detailed description is required.

ANSWER: Thank you for this suggestion. However, our AMT concept included psychoeduction but notpsychotherapy. External applications were used as a self-help technique and served the patients as a method for relaxation and as a support for their self-healing powers. The patients got to know different oils in order to find a suitable self-help for themselves and later applied it independently at home. The aim of *Nutritional counselling* was to give patients education about food intolerances and different food groups and their impact on their microbiota and IBS. The experienced nutritionist did not give advice on specific diets but gave education and answered individual questions in four sessions. The descriptions of all modules have been revised from page 6, line 127 onwards to make them easier to understand.

4. Since psychotherapy and nutritional suggestions have already been shown to be effective in reduction IBS severity, the current results suggested that the AMT by adding anthroposophic components may reverse the proven efficacy of psychotherapy/nutrition suggestions. The authors should comment the limitation on this.

ANSWER: AS our AMT did not include a classical psychotherapy, and also because the subgroup patients with mental comorbidity did not improve after the AMT intervention, our suggestion and conclusion in the manuscript was to apply a psychotherapy for these patients either before AMT or during AMT session. Our study shows that especially for patients without mental comorbidity, the AMT therapy can have a great effect even without explicit psychotherapy. Therefore, it generally seems to make a lot of sense in IBS patients to screen them for their comorbidity and offer adapted therapy, e.g. including psychotherapy in addition to AMT. Lackner et al. (2019) also show that IBS patients with e.g. concurrent anxiety symptoms respond differently to CBT or only educational therapy.

5. Since multimodalities were included in AMT, did all the modalities were required to use by the IBS patients during the 8-week treatment period and follow-up? Was it possible that some IBS patients would prefer some modalities without using other modules, which would lead to the unfavorable outcome in the IBS patients with psychological co-morbidity?

ANSWER: All patients who participated in the study also participated equally in all modalities. Not all patients liked all modules equally (see page 18, line 321). In the satisfaction surveys and oral reports during the therapy, differences in satisfaction with the individual modules were observed. In the explorative statistical analysis, however, no correlations were found between the patients' satisfaction with the AMT therapy and the primary outcome, symptom improvement. These statistical results were not reported due to the focus of the publication and the lack of correlations.
