# Supplementary material for: New multimodal intervention to reduce irritable bowel syndrome (IBS) severity symptoms—Pilot study with a 12 month follow-up
Source: PLoS One. 2022 Nov 21;17(11):e0277880. doi: 10.1371/journal.pone.0277880 (PMC9678296; doi:10.1371/journal.pone.0277880)
Supplement: Attachment — Submitted filename: renamed_04f37.docx [file pone.0277880.s004.docx]

**COMMENTS – Manuscript PONE-D-22-06021**

Title: “New multimodal intervention to reduce Irritable Bowel Syndrome (IBS) severity symptoms - pilot study with a 12 month’s follow up”

**Important note: This review pertains only to ‘statistical aspects’ of the study and so** **‘clinical aspects’ [like medical importance,** **relevance of the study,** **‘clinical significance and implication(s)’ of the whole study, etc.] are to be evaluated [should be assessed] separately/independently. Further please note that any ‘statistical review’ is generally done under the assumption that (such) study specific methodological [as well as execution] issues are perfectly taken care of by the investigator(s). This review is not an exception to that and so does not cover clinical aspects {however, seldom comments are made only if those issues are intimately / scientifically related & intermingle with ‘statistical aspects’ of the study}. Agreed that ‘statistical methods’ are used as just tools here, however, they are vital part of methodology [and so should be given due importance].**

**COMMENTS:** Please note that, your ABSTRACT is well drafted but assay type. It is preferable [refer to item 1b of CONSORT checklist 2010: Structured summary of trial design, methods, results, and conclusions] to divide the ABSTRACT with small sections like ‘Objective(s)’, ‘Methods’, ‘Results’, ‘Conclusions’, etc. which is an accepted practice of most of the good/standard journals [including this one, though ‘The PLoS One Guidelines to Authors’ did not specify an Abstract format, it is desirable]. It will definitely (generally) be more informative then, I guess, whatever the article type may be.

This study being ‘pilot’ in nature, sample size is not a big issue. However, [though many things are ignored (loosely looked at / evaluated)] methodological issues need to be very rigorous followed {in case of clinical trial, CONSORT guidelines are to be strictly observed/followed}. I am sure that the authors are aware of the well-known drawbacks of a single-arm design [a type of Quasi-experimental research], and it is often said that ‘alright to have ‘single-arm design’ (before-after study) when that is the only possibility’, however, it is very essential to keep the limitations in mind while interpreting results. Considering ‘pilot’ (a feasibility study) nature of the study although it is acceptable/alright, as you know, a classical/ideal clinical trial/study needs/requires a concurrently {but similarly} handled/treated appropriately selected/chosen control/comparison parallel group/arm. In this context, please read the following which is pasted from one standard textbook on ‘Research Methodology’:

“Inferential statistics (i.e., hypothesis testing + estimation of CI) is built on the population model [which means the underlying assumption is that there is/are population(s) and we are dealing with random sample(s) drawn from that/those population(s)]. Although in clinical trial (involving at least two groups) we do not really deal with random samples (generally a non-probabilistic convenience sampling), ‘allocation’ to treatment groups is ‘randomly’ done which enable us to evoke the population model and we can use inferential statistics safely. But when there is only one group (so that there is no question of random allocation), with ‘non-random’ selection, it may be questionable to use inferential statistics even if you have two measurement sets as ‘pre-post’ or use ‘internal grouping for comparison.”

Therefore, I guess, it would have been nice to adhered to original plan [lines 89-90] of ‘a waiting group design’.

Although all statistical analyses are performed correctly, few table values [example: q-values (false discovery rate correction for multiple testing) in table-2]] are not explained adequately, in my opinion. How to read [and interpret] these table values may be very briefly explained. Same with table-3.

Please note that the measures/tools used are definitely appropriate, but most of them yield data that are in [at the most] ‘ordinal’ level of measurement [and not in ratio level of measurement for sure. Then application of suitable non-parametric test(s) is/are indicated/advisable [even if distribution may be ‘Gaussian’ (i.e. normal)]. Agreed that there is/are no non-parametric test(s)/technique(s) available to be used as alternative in all situation(s) [suitable / most desired/applicable], but should be used whenever/wherever they are available [for example, instead of paired ‘t’ test use Wilcoxon’s Signed Ranked test].

Moreover, limitations (if any) of the study are not mentioned/listed anywhere. Does that mean {according to authors} there are none? As pointed out in ‘important note’ above “This review pertains only to ‘statistical aspects’ of the study and so ‘clinical aspects’ should be assessed separately/independently [one should carefully consider/look at the clinical implications of the study].

There is no problem in accepting this article after minor revision, in my opinion.
